# Comparison of Cricket Protein Powder and Whey Protein Digestibility

**DOI:** 10.3390/molecules29153598

**Published:** 2024-07-30

**Authors:** Barbora Lampová, Ivo Doskočil, Petr Šmíd, Lenka Kouřimská

**Affiliations:** 1 Department of Microbiology, Nutrition and Dietetics, Faculty of Agrobiology, Food and Natural Resources, Czech University of Life Sciences Prague, Kamycka 129, 165 00 Prague, Czech Republic; doskocil@af.czu.cz (I.D.); kourimska@af.czu.cz (L.K.); 2 Department of Chemistry, Faculty of Agrobiology, Food and Natural Resources, Czech University of Life Sciences Prague, Kamycka 129, 165 00 Prague, Czech Republic; smidp@af.czu.cz

**Keywords:** enthomophagy, insect protein, whey protein, protein digestibility, suitability

## Abstract

With the global population projected to reach nine billion by 2050, the search for alternative protein sources has become critical. This study evaluated the digestibility of cricket protein powder compared with that of whey protein powder. Cricket protein powder had a slightly lower protein content but higher fat content than whey protein powder. Although both contained all essential amino acids, their quantities varied. The most abundant essential amino acid was leucine in both samples. The essential amino acid index (EAAI) for cricket protein powder reached 79% when utilising crude protein for calculation. When using the amino acid sum calculation method, it increased by nearly 13%. The EAAI for whey protein was then 94% when calculated based on crude protein, with a slight increase observed when using the amino acid sum calculation method. Cricket protein exhibited a gradual increase in digestibility during intestinal digestion, reaching nearly 80%, whereas whey protein digestibility surpassed 97%. Despite the lower digestibility of cricket protein compared with whey protein, it remains sufficiently high for consideration as a valuable protein source. This study highlights the potential of cricket proteins and underscores the importance of assessing their protein content and digestibility in evaluating their nutritional value.

## 1. Introduction

The world’s population is growing rapidly, with estimates suggesting that it will reach 9 billion by 2050 [1]. This demographic trend is accompanied by an increase in food demand, presenting a global challenge, especially concerning the provision of an adequate supply of quality proteins [2]. Proteins are key elements for tissue formation and regeneration, as well as optimal human metabolism. To ensure global protein availability, it is necessary to explore new sources of this nutrient [3]. One promising solution to this issue is the consumption of edible insects, also known as entomophagy [4]. Insects are a potential new source of high-quality protein for human nutrition. This option not only helps meet the growing needs of the population but may also be a more environmentally sustainable alternative to traditional food-production methods [5]. This environmentally friendly approach to protein production offers hope for a sustainable future food system, especially considering the growing world population and limited resources [3,5]. Integrating the consumption of edible insects into dietary habits and industrial processes could be a key step towards efficient, sustainable and balanced food production [5]. 

Despite historical mentions of insect consumption, topics related to entomophagy have only recently begun attracting global attention [6]. Insect consumption has become a new trend in food science since 2013 when the Food and Agriculture Organization of the United Nations released a document titled “Edible Insects: Future Prospects for Food and Nutritional Security”. It is estimated that up to 33% of consumed proteins will come from alternative sources by 2054, representing 311 million metric tons of alternative proteins [7]. This trend is further supported by the increasing demand for edible insects, which has sharply increased owing to the transition to high-protein foods. However, the overall protein content of foods does not provide a complete picture of their nutritional quality; the biological availability of specific nutrients is a key parameter that must be considered [8]. The biological availability of a nutrient is defined as the fraction that is soluble in the gastrointestinal environment and available for absorption. Biological availability is expected to vary among insect species [9]. However, most species typically have a high protein digestibility, ranging from 80% to 90% [10].

Notably, insects are not traditional foods in Western societies and despite their increasing demand, they are often associated with resistance or neophobia in these societies. However, it has been found that attitudes towards different insect products vary. For example, attitudes towards whole insects are less favourable than those toward processed insect products. Therefore, the visibility of insects in food is a crucial factor directly influencing the acceptance of insect-based foods. A suitable option for incorporating insects into human food is to process them into insect powders, which have wide applications in the baking industry [11]. Bakery products are commonly made from wheat flour, which means that they are high in energy but poor in nutrients [12]. In addition, the protein quality of wheat flour is low because of the presence of limited essential amino acids, especially lysine [13]. To improve the nutritional profile of these basic foods, especially the content and quality of proteins, fibre and minerals, the trend of enriching bakery products with edible insects has recently been followed. Cricket powder is very often used for these purposes [14]. The nutritional composition of various cricket species often rivals that of traditional livestock meats and staple crops, both in terms of quantity and quality. Crickets are recognised for their rich nutrient profile, which includes high-quality proteins that are readily digestible and highly bioavailable compared to nutrients derived from conventional plant and animal sources. For instance, *Acheta domesticus* demonstrates impressive protein digestibility, ranging from 84% to 92% without prior heating and potentially up to 90% following heat treatment. These findings underscore the suitability of crickets as a sustainable and nutritious alternative protein source in modern diets [15,16]. Although data on the digestibility of various edible insect species are already available in the literature, relatively few data exist on the digestibility of edible insect-based products [17].

The aim of this study was to assess the quality of cricket protein powder intended for baking and cooking by determining the essential amino acid index and total digestibility of the protein. Unlike previous studies that have primarily assessed the nutritional content of whole insects, a more comprehensive understanding of how cricket protein behaves during digestion when incorporated into food products was sought. To achieve this, the digestibility of cricket protein was compared with that of whey protein. Whey protein was selected for comparison because it is a well-established, highly digestible protein source with an absorption rate of approximately 100%. The development of digestibility during the intestinal phase of digestion was monitored using an in vitro digestion model. By comparing these two proteins, novel insights into the practical applications of cricket protein in enhancing the nutritional quality of everyday foods are offered. The findings will contribute to the growing body of knowledge on edible insects and support their potential as a viable alternative in the global protein supply chain [18,19].

## 2. Results

In this study, we assessed the digestibility of commercially available cricket protein powder as a novel source of sustainable proteins of animal origin. Subsequently, the digestibility of the cricket protein powder was compared with that of whey protein, which is renowned as one of the best and most rapidly digestible proteins.

### 2.1. Nutritional Value

In this study, statistically significant differences were found between the protein (F = 346.3; *p* < 0.0001) and fat contents (F = 1152; *p* < 0.0001) of cricket and whey protein samples. Cricket protein powder contained approximately 10% less protein than whey protein and 15% more fat than whey protein. Both samples contained all the analysed amino acids, but their contents varied significantly between the samples (*p* < 0.003 for proline, *p* < 0.02 for valine, *p* < 0.005 for phenylalanine, *p* < 0.0001 for other amino acids). Cricket proteins had the highest alanine and the lowest threonine content. Cricket protein had the highest content of leucine among all the essential amino acids present. Whey protein had the highest overall glutamic acid content and the lowest glycine content. Like cricket protein powder, whey protein had the highest leucine content among the essential amino acids. However, it should be noted that the full spectrum of amino acids was not determined, and tryptophan was not measured among the essential amino acids (Table 1). The calculated values of essential amino acids (EAAs) and the essential amino acid index (EAAI) for cricket protein powder and whey protein are presented in Table 2. The limiting amino acid for cricket protein powder was isoleucine, whereas for whey protein, phenylalanine. The EAAI differed substantially between the two samples, with cricket protein powder reaching approximately 20% lower values than whey protein when calculated based on CP. Calculations based on the sum of amino acids showed that cricket protein powder increased EAAI by nearly 13%.

### 2.2. Protein Digestibility

Table 3 and Table 4 show the results of amino acid digestibility during intestinal phase of in vitro digestion in both tested samples. The digestibility of individual amino acids increased throughout the entire intestinal digestion phase. The highest increase in digestibility occurred within the first 40 min of intestinal digestion. The overall digestibility of cricket protein powder (Table 3) was slightly above 50% at the beginning of the intestinal digestion phase and increased to nearly 80% during this phase, with a 20% increase in digestibility during the first 40 min of intestinal digestion and a further increase of just under 8% in the following 80 min. The overall digestibility of whey protein (Table 4) at the beginning of the intestinal digestion phase was almost 10% higher than that of cricket protein powder, and it increased to nearly 100% during this phase. Similar to cricket protein, there was a 20% increase in digestibility during the first 40 min of intestinal digestion, but in the following 80 min, the increase in digestibility was higher than that of cricket protein powder, although not as high as in the first 40 min of intestinal digestion.

## 3. Discussion

Protein was the dominant component in the cricket protein sample. This finding was also confirmed in the study by Ververis et al. (2022), where the protein content in *Acheta domesticus* per dry matter ranged from 41.8 g/100 g to 75.2 g/100 g [20]. Our observed value was near the higher limit of this range and was even by 2.4 g/100 g higher than that stated on the product label. Several factors could contribute to the differences in protein content between our findings and those of Ververis et al. (2022). One possible reason could be the diet and rearing conditions of the crickets. Different types of feed can significantly impact the nutritional composition of the insects. Crickets fed on high-protein diets may have higher protein contents compared to those fed on more carbohydrate-rich diets. Environmental factors such as rearing temperature, humidity, and the age at which the crickets are harvested can also influence their protein levels. Another factor could be protein content determination method [21]. Protein content in our study was determined using the Kjeldahl method, which determines the total nitrogen (TN) content and is subsequently multiplied by a conversion factor. In our study, a universal conversion factor of 6.25 was used. However, this approach may be problematic for insect analyses. Insects contain a high proportion of non-protein nitrogen compounds, such as chitin, nucleic acids, phospholipids, etc. These components may lead to an overestimation of the protein content because the Kjeldahl method does not distinguish between nitrogen from proteins and nitrogen from non-proteinaceous sources. This was further supported by the sum of the determined amino acids, which indicated a protein content approximately 10% lower than the protein content determined as TN multiplied by a conversion factor of 6.25. By using the conversion factor of 5.09, which is recommended by Ritvanen et al. (2020) for *Acheta domesticus*, an even lower protein content of 58.97 ± 0.38 g/100 g DM was achieved [22]. This value approached much closer to the value determined by the sum of the amino acids. The most abundant amino acids in cricket protein samples found in our study were alanine, glutamic acid and aspartic acid, each with a content exceeding 5 g/100 g dry matter. Similarly, in the study by Ververis et al. (2022), all of the mentioned amino acids exceeded 5 g/100 g of the sample [20]. The same results were also reached in the study by Stone et al. (2019) [23]. 

The whey protein analysed in our study was derived from cow milk and contained slightly more than 80% protein and roughly 10% more protein than that determined in the cricket protein sample. Similarly, for this product, the protein content determined by us was slightly higher than that stated by the manufacturer on the product label. The sum of amino acids in the whey protein resembled the protein content determined using TN multiplied by a conversion factor of 6.38. Similar results for protein content were also found in a study by Almeida et al. (2015), in which 15 commercially available whey proteins were analysed [24]. The content of individual amino acids differed among the analysed samples. Glutamic acid was the most abundant amino acid, with its content being more than double that of the cricket protein sample.

Like other food sources, a product’s nutrient content alone is not sufficient to assess the nutritional value. It is important to assess the quality of a given nutrient. In the case of proteins, the key factors in evaluation are the amino acid score (AAS) and EAAI. A protein source with an EAAI exceeding 95 signifies superiority, whereas an EAAI ranging from 86 to 95 indicates a good protein source. A source with an EAAI ranging from 76 to 86 is considered usable, whereas an EAAI value below or equal to 75 indicates an unsuitable protein source [25].

In our study, cricket protein demonstrated an EAAI 79.31% when calculated using the CP method, indicating its classification as a usable protein source. However, because of the likelihood of overestimation of protein content inherent in CP calculations with a conversion factor of 6.25, we conducted additional calculations based on the sum of the amino acids. This approach was chosen because of its potential to provide a more accurate reflection of true protein content, avoiding the potential underestimation of the EAAI associated with the CP method. The initial EAAI values for cricket protein were initially below 80% when calculated using the CP method (with a conversion factor of 6.25). However, these values significantly rose to approximately 92% when computed based on the sum of amino acids. This adjustment resulted in a notable increase of approximately 13% in the EAAI, thereby upgrading the classification of cricket proteins to that of a good protein source. Of particular interest is the comparison of the performance of cricket protein with that of whey protein in amino acid sum-based calculations. Whey protein has already achieved an EAAI exceeding 90% when calculated using the CP method, with a minimal further increase observed when utilising the sum of amino acids. When calculating EAAI based on the sum of amino acids, a method that offers greater accuracy in assessing protein content in edible insect samples, cricket protein powder achieved an EAAI value only 2% lower than that of whey protein. This observation suggests a comparable quality profile between cricket and whey proteins, despite differences in their initial EAAI values derived from CP calculations.

The second key factor is the determination of protein digestibility [26]. In the literature, many factors influence digestibility, including digestion methodology, result evaluation and species-specific characteristics or developmental stages. Although these factors may vary, the digestibility of insect proteins is generally high, with most species exhibiting digestibility between 80 and 90%. The total protein digestibility of the cricket protein samples in our study was almost 80%, which is consistent with previous findings [10]. Similarly, Ndiritu et al. (2017) found high protein digestibility in *Acheta domesticus* ranging from 84.23 to 85.28 depending on the type of extraction used [27]. Conversely, low digestibility was found in the study by Poelaert et al. (2016); however, it should be noted that this research focused on protein digestibility in pigs, which may have led to different results from those of our study. The discrepancy in in vitro protein digestibility between insects and pigs could be attributed to several factors. These factors include differences in digestive physiology and enzyme activity between the two species, variations in digestion methodology and evaluation criteria used in studies [28].

In our study, a high digestibility of whey protein was observed, exceeding 97% by the end of the intestinal phase. Slightly lower results were obtained in the study by Almeida et al. (2015), in which the digestibility of eight commercially available whey proteins from American companies and seven whey proteins from Brazil were analysed. The digestibility of whey proteins in the American market was 91.7%, whereas that of whey proteins from Brazil is 3.3% lower [24]. Therefore, whey protein provided higher digestibility at all time points of intestinal digestion compared to the cricket protein sample by approximately 10–25%. This difference could be attributed to the higher fibre content in insect proteins, where the manufacturer declares 10% fibre (Table 5). Fibre content is negatively correlated with protein digestibility [29]. However, fibre content may not be a negative criterion for inclusion in the diet, as fibre is typically lacking in the human diet and has a positive impact on gastrointestinal health and the microbiome. Moreover, fat increases the energy density of food [30,31]. The lower digestibility of the selected cricket protein samples may also be due to the choice of the samples. The selected cricket protein sample is mainly intended for cooking and baking, unlike whey protein, which serves as a fast and easily digestible source of protein after exercise [32]. 

Determining the overall digestibility of proteins is important; however, this only considers the absorbed percentage of proteins. It is essential to consider the content of essential amino acids as they are crucial for the proper functioning of the human body and tissue growth. Without adequate amounts of essential amino acids, a high percentage of absorbed proteins may be ineffective for nutrition. In addition, the digestibility of individual amino acids can be compared to each other. Therefore, in the present study, the digestibility of individual amino acids was determined. The digestibility of individual amino acids varied within a given sample, but not significantly. For the cricket protein sample, the digestibility of individual amino acids in the final stage of digestion ranged from 77.86 to 82.78%, with the lowest digestibility achieved for tyrosine and the highest for proline. A very important finding was that all essential amino acids reached a digestibility higher than 78% at the end of intestinal digestion. In the case of whey protein, the digestibility of individual amino acids in the final stage of intestinal digestion ranged from 97.85 to 99.26%, with leucine exhibiting the lowest digestibility and proline showing the highest digestibility, similar to that of the cricket protein sample.

Analysing the time-dependent digestion of amino acids provides important insights into the efficiency of nutrient absorption from different protein sources. The results demonstrated that both cricket and whey proteins showed significant increases in digestibility during the first 40 min of intestinal digestion, which indicates a rapid initial breakdown and absorption of amino acids. Specifically, the cricket protein powder experienced a 20% increase in digestibility in this period, followed by a more gradual increase over the next 80 min, resulting in an overall digestibility close to 80%. In comparison, the whey protein also showed a 20% increase in the first 40 min, but continued to absorb more efficiently over the remaining 80 min, nearing complete absorption at nearly 100%.

This time-dependent pattern suggests that while both proteins are rapidly digested initially, whey protein maintains a higher absorption rate throughout the digestion process. This can be attributed to the inherent properties of whey protein, which include a high proportion of easily digestible amino acids and low non-protein nitrogen content. These factors enable whey protein to be utilised more efficiently by the body, supporting quicker muscle recovery and growth, which is particularly beneficial post-exercise. Conversely, the presence of fibre in cricket protein, although beneficial for gastrointestinal health, may slow down the complete digestion of amino acids [18,30,31].

Although cricket protein powder achieved a lower digestibility than whey protein, its digestibility can still be considered high. In addition, a great advantage of cricket powder is its fibre content, which is insufficiently consumed by the European population. Another significant advantage is the ecological profile of cricket powder. The production of cricket powder has a significantly lower ecological footprint than traditional animal sources of protein, including whey protein, as cricket powder requires less water, feed and land. This factor promotes sustainability and can help address some of the global challenges associated with food production.

## 4. Materials and Methods

### 4.1. Samples

For the purposes of this study, two different commercially available protein-rich samples were used: protein powder made from house crickets (*Acheta domesticus*) (Cricket protein powder for cooking and baking SENS FOODS, SENS Foods, the United Kingdom of Great Britain and Northern Ireland) and whey protein powder (Pure Whey Protein Bulk, www.bulkpowders.com (accessed on 4 October 2022), the United Kingdom of Great Britain and Northern Ireland). The proximate nutritional composition is presented in Table 5.

### 4.2. Nutrient Analysis

A nutritional assessment was performed three times for each sample. The dry matter percentage was assessed through a drying process in an oven set at 103 ± 2 °C until a constant weight was achieved. Nitrogen levels were measured using the Kjeldahl method based on ISO 5983-1:2005 [33] in a Kjeltec 2400 analyser (FOSS, Hillerød, Denmark). The crude protein content was then calculated using a nitrogen-to-protein conversion factor of 6.25 for the cricket protein sample and 6.38 for the whey protein.

Amino acid profiles were determined according to ISO 13903:2005 standard [34]. Sample preparation involved oxidative hydrolysis of sulfur-containing amino acids and acid hydrolysis of other amino acids. The sample (0.5 g) was mixed with a few drops of ethanol (Penta, Prague, Czech Republic) and 50 mL of 6 M hydrochloric acid (Lach-Ner, Neratovice, Czech Republic). Subsequently, the sample was purged with nitrogen (Linde, Prague, Czech Republic) and hydrolysed in closed glass tubes for 23 h at 110 °C.

For oxidative hydrolysis, the sample (0.5 g) was mixed with 5–15 mL of an oxidative mixture containing hydrogen peroxide and formic acid at a ratio of 1:9 (Lach-Ner, Neratovice, Czech Republic). The mixture was refrigerated for 16 h. Subsequently, 100 mL of 6 M hydrochloric acid (Lach-Ner, Neratovice, Czech Republic) was added to the sample, and the entire hydrolytic reaction was conducted for 23 h on a heating plate with the temperature controlled to achieve gentle boiling.

Hydrolysed samples were subsequently filtered and evaporated using a vacuum evaporator (Heidolph Instruments GmH & Co., Schwabach, Germany) at 50 °C. They were then analysed using an amino acid analyser 400 (INGOS, Prague, Czech Republic) with sodium citrate buffer and hydrolysate standards CYS-H/MET-S (INGOS, Prague, Czech Republic). The analytical process included post-column ninhydrin derivatisation and spectrophotometric detection. Tryptophan was not detected because of its decomposition during acid hydrolysis.

AAS was calculated by comparing the concentration of each essential amino acid (EAA) in the test protein with the concentration of the same amino acid in a reference protein according to Equation (1). As a reference whole egg protein was used.

Equation (1): Amino acid score (AAS)

(1) $$AAS=\frac{EAA}{{EAA}_{ref}}\times 100$$

where EAA is concentration of the essential amino acid in the tested sample (mg/g of protein) and EAA_ref_ is concentration of the essential amino acid in the reference protein (mg/g of protein).

The EAAI was derived as the geometric average of the AAS values for all essential amino acids according to Equation (2).

Equation (2): Essential amino acid index (EAAI)

(2) $$EAAI=\sqrt[{n}]{\prod_{i=1}^{n}\left(\frac{{EAA}_{i}}{{EAA}_{ref,i}}\right)\times 100}$$

where *n* is number of essential amino acids; EAA_i_ is concentration of the i-th essential amino acid in the tested sample (mg/g of protein) and EAA_ref,i_ is concentration of the i-th essential amino acid in the reference protein (mg/g of protein).

### 4.3. Static In Vitro Digestion Model

**Figure 1:**
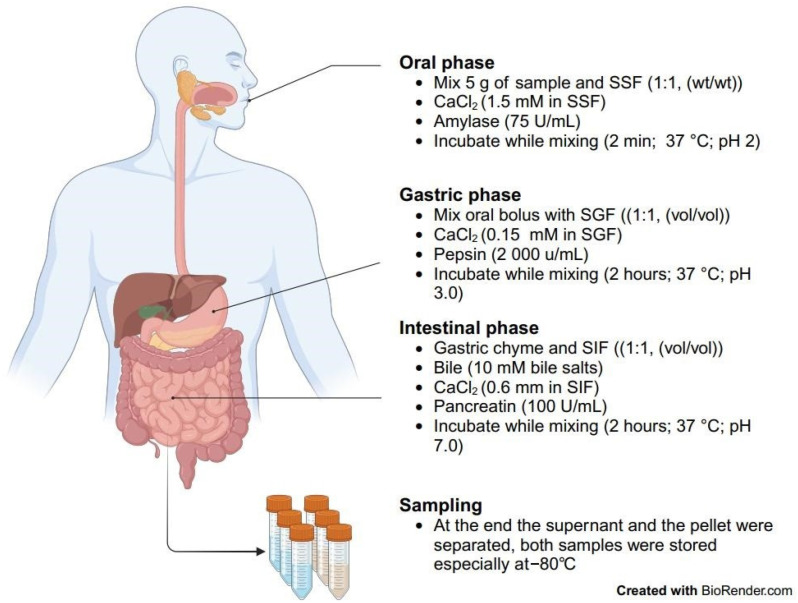
Flow diagram of the in vitro digestion model.

An in vitro static digestion model was simulated as described by Brodkorb et al. (2019) [35]. The process is illustrated in the flow diagram of the in vitro digestion model (Figure 1). 

In summary, 5 g of the specimen was combined with 5 mL of simulated saliva solution (15.1 mM KCl; 3.7 mM KH_2_PO_4_; 13.6 mM NaHCO_3_; 0.15 mM MgCl_2_·6H_2_O; 0.06 mM (NH_4_)_2_CO_3_; 1.1 mM HCl; 1.5 mM CaCl_2_·2H_2_O) and amylase (75 U/mL in total digestate; Sigma-Aldrich s.r.o., St. Louis, MO, USA). The mixture was incubated for 2 min at 37 °C and pH 7.

Following this, the oral bolus was combined with 10 mL of simulated gastric fluid (6.9 mM KCl; 0.9 mM KH_2_PO_4_; 25 mM NaHCO_3_; 47.2 mM NaCl; 0.12 mM MgCl_2_·6H_2_O; 0.5 mM (NH_4_)_2_CO_3_; 15.6 mM HCl; 0.15 mM CaCl_2_·2H_2_O), along with pepsin (2000 U/mL in total digestate; Sigma-Aldrich s.r.o.). The mixture was incubated for 120 min at 37 °C and pH 3. This pH was maintained constant throughout the entire gastric phase of digestion.

Subsequently, the gastric chyme was mixed with 15 mL of simulated intestinal fluid (6.8 mM KCl; 0.8 mM KH_2_PO_4_; 85 mM NaHCO_3_; 38.4 mM NaCl; 0.33 mM MgCl_2_·6H_2_O; 8.4 mM HCl; 0.6 mM CaCl_2_·2H_2_O), bile (10 mM in total digestate; Sigma-Aldrich s.r.o.) and pancreatin (trypsin activity 100 U/mL in total digestate; Sigma-Aldrich s.r.o.). The mixture was incubated for 120 min at 37 °C and pH 7. This pH was maintained constant throughout the entire intestinal phase of digestion. The digestion process was halted by freezing the samples at −80 °C.

Samples were collected every 20 min from the beginning of the intestinal digestion phase until completion.

### 4.4. Determination of Protein Digestibility

After digestion, the samples were centrifuged for 10 min at 3500× *g* to separate the undigested proteins from the amino acids (digested parts). Total digestibility was calculated by comparing the amino acid content in the undigested and digested samples according to Equation (3).

Equation (3): In vitro protein digestibility was calculated from the sum of amino acids (AA) in the digested and undigested samples.

(3) $$Total\;digestibility\left(\%\right)=\frac{AA\;in\;digested\;samples}{AA\;in\;undigested\;samples}\times 100$$

### 4.5. Statistical Analysis

The data underwent statistical analysis using multi-way analysis of variance (ANOVA), followed by post-hoc tests using Scheffe’s method, with a significance level set at α = 0.05. Statistical analysis was conducted using Statistica 13.2 software package (StatSoft, Inc., Tulsa, OK, USA). F-values were computed to assess the differences between groups. The findings are reported as arithmetic means (x) accompanied by their respective standard deviations derived from observations across three distinct sample sets.

## 5. Conclusions

This study evaluated the quality of cricket protein powder by determining the EAAI and protein digestibility. The obtained results were subsequently compared with whey protein, a well-established protein known for its rapid digestion and absorption. Two calculation methods were chosen to calculate the EAAI. The method using the protein content determined as crude protein with a conversion factor of 6.25 gave a lower value than the calculation method using the sum of amino acids. The second method then gave an EAAI higher than 90%, a result comparable to whey protein. Our study found that cricket protein powder showed a digestibility rate of almost 80%, whereas whey protein showed substantially higher digestibility, exceeding 97%. Despite the lower digestibility of cricket protein powder compared to whey protein, it remains high enough to be considered a valuable protein source. This difference in digestibility could potentially be attributed to the higher fibre content of the cricket protein powder. However, it should be noted that this fibre content may also have beneficial effects on gastrointestinal health and overall nutritional value.

Future research should focus on enhancing the digestibility of cricket protein powder. Investigating the effects of different processing methods, such as fermentation or enzymatic treatment, could help break down chitin and other fibrous components that may inhibit protein absorption. Additionally, exploring the impact of blending cricket protein with other plant or animal proteins might improve its overall digestibility and amino acid profile. Long-term studies on the effects of regular consumption of cricket protein on human gastrointestinal health and nutrient absorption will also be beneficial. Finally, in vivo studies are needed to validate the findings from in vitro digestibility assays and to understand the bioavailability of essential amino acids from cricket protein in the human body.

## Figures and Tables

**Table 1:** Basic nutritional parameters and amino acid content of the dry matter of cricket and whey protein samples.

Component (g/100 g DM)	Cricket Protein Powder	Whey Protein
Crude protein (TN × 6.25)	72.41 ± 0.46 ^b^	82.89 ± 0.49 ^a^
Total fat	18.35 ± 0.53 ^b^	4.80 ± 0.23 ^a^
Aspartic acid	5.25 ± 0.11 ^b^	7.72 ± 0.11 ^a^
Threonine	2.58 ± 0.12 ^b^	5.21 ± 0.16 ^a^
Serine	2.85 ± 0.11 ^b^	5.04 ± 0.11 ^a^
Glutamic acid	5.60 ± 0.15 ^b^	12.59 ± 0.12 ^a^
Proline	3.81 ± 0.12 ^b^	4.49 ± 0.14 ^a^
Glycine	3.23 ± 0.13 ^a^	2.09 ± 0.12 ^b^
Alanine	6.27 ± 0.17 ^a^	3.98 ± 0.17 ^b^
Valine	4.03 ± 0.15 ^b^	4.49 ± 0.15 ^a^
Isoleucine	2.93 ± 0.11 ^b^	4.75 ± 0.12 ^a^
Leucine	5.14 ± 0.14 ^b^	7.72 ± 0.10 ^a^
Tyrosine	4.09 ± 0.13 ^a^	2.77 ± 0.12 ^b^
Phenylalanine	2.93 ± 0.09 ^a^	2.43 ± 0.12 ^b^
Methionine	2.83 ± 0.13 ^b^	2.84 ± 0.13 ^a^
Histidine	2.73 ± 0.10 ^b^	6.18 ± 0.09 ^a^
Lysine	4.07 ± 0.12 ^b^	7.61 ± 0.12 ^a^
Arginine	3.93 ± 0.12 ^a^	2.30 ± 0.13 ^b^
Sum of AA	62.29 ± 1.62 ^b^	82.21 ± 1.64 ^a^

AA, amino acids; DM, dry matter; TN, total nitrogen. Values are expressed as means ± standard deviations (*n* = 3); numbers on the same raw followed by different lowercase letters are statistically different (*p* ≤ 0.05).

**Table 2:** Amino acid score (AAS) and essential amino acid index (EAAI) of cricket protein powder and whey protein.

Sample	Cricket Protein Powder		Whey Protein	
	Crude Protein	Sum of AA	Crude Protein	Sum of AA
Threonine	69.99 ± 2.58 ^c^	81.36 ± 3.00 ^b^	123.30 ± 3.09 ^a^	124.32 ± 3.12 ^a^
Valine	76.25 ± 2.32 ^b^	88.63 ± 2.69 ^a^	74.16 ± 2.02 ^b^	74.77 ± 2.04 ^b^
Isoleucine	61.31 ± 1.82 ^c^	71.27 ± 2.12 ^b^	86.86 ± 1.79 ^a^	87.58 ± 1.81 ^a^
Leucine	80.62 ± 1.84 ^c^	93.72 ± 2.14 ^b^	105.85 ± 1.08 ^a^	106.72 ± 1.09 ^a^
Phenylalanine	69.77 ± 1.81 ^b^	81.10 ± 2.11 ^a^	50.50 ± 2.04 ^c^	50.92 ± 2.05 ^c^
Methionine	102.13 ± 4.58 ^b^	141.98 ± 5.33 ^a^	107.07 ± 4.00 ^b^	107.96 ± 4.03 ^b^
Lysine	87.82 ± 2.17 ^c^	102.09 ± 2.53 ^b^	143.37 ± 1.80 ^a^	144.55 ± 1.81 ^a^
EAAI (%)	79.31 ± 2.36 ^b^	92.19 ± 2.74 ^a^	94.06 ± 2.33 ^a^	94.84 ± 2.35 ^a^

AA: amino acid; EAAI: essential amino acid index. Values are expressed as means ± standard deviations (*n* = 3); numbers on the same raw followed by different lowercase letters are statistically different (*p* ≤ 0.05).

**Table 3:** Digestibility of amino acids during the intestinal phase of in vitro digestion of cricket protein samples.

Amino Acid Digestibility (%)	Time of Intestinal Phase In Vitro Digestion						
	0 min	20 min	40 min	60 min	80 min	100 min	120 min
Aspartic acid	52.03 ± 0.17 ^f^	65.48 ± 0.15 ^e^	73.00 ± 0.16 ^d^	73.02 ± 0.16 ^d^	73.61 ± 0.17 ^c^	75.17 ± 0.13 ^b^	80.40 ± 0.18 ^a^
Threonine	49.63 ± 0.15 ^f^	63.99 ± 0.18 ^e^	70.71 ± 0.17 ^d^	70.78 ± 0.15 ^d^	71.42 ± 0.17 ^c^	72.91 ± 0.19 ^b^	78.84 ± 0.17 ^a^
Serine	49.39 ± 0.20 ^e^	64.03 ± 0.19 ^d^	71.24 ± 0.20 ^c^	71.42 ± 0.18 ^c^	71.76 ± 0.17 ^c^	73.33 ± 0.20 ^b^	78.92 ± 0.20 ^a^
Glutamic acid	53.05 ± 0.20 ^e^	66.67 ± 0.19 ^d^	73.45 ± 0.20 ^c^	73.73 ± 0.18 ^c^	73.99 ± 0.20 ^c^	75.06 ± 0.21 ^b^	80.31 ± 0.22 ^a^
Proline	58.78 ± 0.18 ^e^	70.99 ± 0.16 ^d^	75.87 ± 0.17 ^c^	76.64 ± 0.18 ^b^	76.59 ± 0.19 ^b^	77.04 ± 0.18 ^b^	82.78 ± 0.17 ^a^
Glycine	51.92 ± 0.20 ^e^	65.97 ± 0.22 ^d^	70.59 ± 0.21 ^c^	70.63 ± 0.22 ^c^	71.28 ± 0.20 ^c^	72.88 ± 0.23 ^b^	78.32 ± 0.23 ^a^
Alanine	52.87 ± 0.21 ^f^	66.51 ± 0.23 ^e^	71.13 ± 0.22 ^d^	71.42 ± 0.21 ^cd^	72.02 ± 0.23 ^c^	73.90 ± 0.22 ^b^	78.67 ± 0.23 ^a^
Valine	40.05 ± 0.19 ^f^	68.14 ± 0.21 ^e^	71.65 ± 0.19 ^d^	72.64 ± 0.21 ^c^	72.89 ± 0.22 ^bc^	73.37 ± 0.21 ^b^	79.27 ± 0.20 ^a^
Isoleucine	50.61 ± 016 ^f^	65.54 ± 0.18 ^e^	71.04 ± 0.17 ^d^	71.70 ± 0.18 ^c^	72.78 ± 0.18 ^b^	72.84 ± 0.19 ^b^	78.91 ± 0.18 ^a^
Leucine	50.68 ± 0.19 ^e^	66.49 ± 0.20 ^d^	70.89 ± 0.21 ^c^	71.40 ± 0.21 ^c^	71.44 ± 0.19 ^c^	73.22 ± 0.18 ^b^	78.26 ± 0.20 ^a^
Tyrosine	48.31 ± 0.18 ^f^	63.09 ± 0.19 ^e^	69.28 ± 0.21 ^d^	69.32 ± 0.20 ^d^	70.40 ± 0.18 ^c^	71.46 ± 0.18 ^b^	77.86 ± 0.19 ^a^
Phenylalanine	53.81 ± 0.17 ^f^	65.75 ± 0.17 ^e^	72.03 ± 0.14 ^d^	72.51 ± 0.16 ^d^	73.77 ± 0.15 ^c^	74.83 ± 0.15 ^b^	80.56 ± 0.13 ^a^
Methionine	52.10 ± 0.17 ^f^	66.53 ± 0.18 ^e^	72.1 ± 0.18 ^d^	72.36 ± 0.20 ^cd^	72.94 ± 0.19 ^c^	74.35 ± 0.21 ^b^	79.65 ± 0.17 ^a^
Histidine	57.16 ± 0.17 ^g^	66.34 ± 0.16 ^f^	69.66 ± 0.17 ^e^	71.31 ± 0.16 ^d^	71.88 ± 0.15 ^c^	74.87 ± 0.15 ^b^	79.35 ± 0.16 ^a^
Lysine	58.60 ± 0.18 ^f^	70.28 ± 0.18 ^e^	75.16 ± 0.18 ^d^	75.36 ± 0.17 ^cd^	75.90 ± 0.16 ^c^	78.13 ± 0.16 ^b^	81.97 ± 0.17 ^a^
Arginine	54.84 ± 0.18 ^f^	67.11 ± 0.19 ^e^	72.48 ± 0.17 ^d^	72.49 ± 0.17 ^d^	73.38 ± 0.18 ^c^	75.50 ± 0.17 ^b^	79.94 ± 0.19 ^a^
Total digestibility (%)	52.11 ± 2.14 ^c^	66.54 ± 2.18 ^b^	72.00 ± 2.18 ^ab^	72.38 ± 2.14 ^ab^	72.93 ± 2.17 ^ab^	74.37 ± 2.17 ^ab^	79.64 ± 2.16 ^a^

Values are expressed as means ± standard deviations (*n* = 3); numbers on the same raw followed by different lowercase letters are statistically different (*p* ≤ 0.05).

**Table 4:** Digestibility of amino acids during the intestinal phase of in vitro digestion of whey protein samples.

Amino Acid Digestibility (%)	Time of Intestinal Phase In Vitro Digestion						
	0 min	20 min	40 min	60 min	80 min	100 min	120 min
Aspartic acid	62.93 ± 0.19 ^f^	76.46 ± 0.18 ^e^	81.90 ± 0.17 ^d^	92.46 ± 0.19 ^c^	97.61 ± 0.19 ^b^	98.36 ± 0.17 ^a^	98.81 ± 0.18 ^a^
Threonine	63.07 ± 0.21 ^f^	76.50 ± 0.20 ^e^	81.92 ± 0.21 ^d^	92.38 ± 0.21 ^c^	97.43 ± 0.18 ^b^	97.92 ± 0.20 ^ab^	98.32 ± 0.19 ^a^
Serine	62.66 ± 0.19 ^f^	76.18 ± 0.21 ^e^	81.84 ± 0.19 ^d^	92.33 ± 0.20 ^c^	97.49 ± 0.19 ^b^	97.84 ± 0.18 ^ab^	98.28 ± 0.17 ^a^
Glutamic acid	62.43 ± 0.17 ^f^	75.98 ± 0.18 ^e^	81.50 ± 0.14 ^d^	92.17 ± 0.18 ^c^	97.28 ± 0.18 ^b^	97.67 ± 0.16 ^ab^	98.13 ± 0.17 ^a^
Proline	64.68 ± 0.22 ^f^	76.18 ± 0.23 ^e^	82.23 ± 0.21 ^d^	92.45 ± 0.24 ^c^	98.36 ± 0.24 ^b^	98.54 ± 0.21 ^ab^	99.26 ± 0.23 ^a^
Glycine	62.73 ± 0.19 ^f^	76.28 ± 0.18 ^e^	81.72 ± 0.17 ^d^	92.26 ± 0.18 ^c^	97.50 ± 0.16 ^b^	97.79 ± 0.17 ^ab^	98.19 ± 0.18 ^a^
Alanine	63.61 ± 0.21 ^f^	76.63 ± 0.20 ^e^	82.10 ± 0.21 ^d^	92.58 ± 0.20 ^c^	98.08 ± 0.22 ^b^	98.44 ± 0.23 ^ab^	98.81 ± 0.21 ^a^
Valine	63.71 ± 0.19 ^f^	76.78 ± 0.21 ^e^	82.66 ± 0.19 ^d^	92.64 ± 0.21 ^c^	97.74 ± 0.22 ^b^	98.13 ± 0.21 ^ab^	98.50 ± 0.20 ^a^
Isoleucine	63.02 ± 0.18 ^f^	76.08 ± 0.17 ^e^	81.78 ± 0.16 ^d^	92.35 ± 0.16 ^c^	97.55 ± 0.17 ^b^	97.86 ± 0.18 ^ab^	98.31 ± 0.18 ^a^
Leucine	61.38 ± 0.15 ^f^	75.20 ± 0.16 ^e^	81.02 ± 0.16 ^d^	91.64 ± 0.16 ^c^	96.93 ± 0.13 ^b^	97.32 ± 0.18 ^ab^	97.85 ± 0.17 ^a^
Tyrosine	63.33 ± 0.16 ^f^	76.78 ± 0.18 ^e^	81.80 ± 0.18 ^d^	92.57 ± 0.17 ^c^	97.84 ± 0.18 ^b^	97.84 ± 0.19 ^b^	98.71 ± 0.20 ^a^
Phenylalanine	64.42 ± 0.19 ^f^	77.20 ± 0.18 ^e^	82.73 ± 0.18 ^d^	93.16 ± 0.17 ^c^	98.20 ± 0.18 ^b^	98.55 ± 0.20 ^ab^	98.87 ± 0.18 ^a^
Methionine	60.89 ± 0.18 ^f^	73.61 ± 0.20 ^e^	79.01 ± 0.18 ^d^	89.16 ± 0.19 ^c^	94.16 ± 0.20 ^b^	94.53 ± 0.18 ^ab^	94.96 ± 0.21 ^a^
Histidine	64.83 ± 0.14 ^f^	77.04 ± 0.14 ^e^	82.16 ± 0.17 ^d^	92.60 ± 0.17 ^c^	97.60 ± 0.16 ^b^	97.93 ± 0.15 ^ab^	98.16 ± 0.16 ^a^
Lysine	62.53 ± 0.19 ^f^	75.57 ± 0.17 ^e^	81.75 ± 0.19 ^d^	92.33 ± 0.18 ^c^	97.33 ± 0.18 ^b^	97.74 ± 0.18 ^ab^	98.17 ± 0.20 ^a^
Arginine	63.77 ± 0.18 ^f^	76.75 ± 0.20 ^e^	82.41 ± 0.15 ^d^	92.73 ± 0.18 ^c^	97.81 ± 0.19 ^b^	98.14 ± 0.16 ^ab^	98.52 ± 0.18 ^a^
Total digestibility (%)	63.06 ± 2.18 ^c^	76.23 ± 2.17 ^b^	81.85 ± 2.13 ^b^	92.36 ± 2.18 ^a^	97.54 ± 2.18 ^a^	97.93 ± 2.21 ^a^	98.38 ± 2.19 ^a^

Values are expressed as means ± standard deviations (*n* = 3); numbers on the same raw followed by different lowercase letters are statistically different (*p* ≤ 0.05).

**Table 5:** Nutritional composition of the tested samples as declared by the manufacturer.

Nutrition Information per 100 g		
	Cricket Protein Powder	Pure Whey Protein
Energy value	1939 kJ/463 kcal	367 kJ/1554 kcal
Fat (g)	20	5.0
of which saturated fatty acids (g)	5.2	0.3
Carbohydrates (g)	0.5	3.5
of which sugars (g)	0	3.5
Fibre (g)	9.5	0
Protein (g)	70	80
Salt (g)	0.8	0.51

## Data Availability

Data will be made available on request.
